# Urinary Exosomes as Potential Biomarkers for Diagnosis, Disease Activity Assessment, Treatment Monitoring, and Prognosis in Lupus Nephritis

**DOI:** 10.3390/ijms27157051

**Published:** 2026-08-06

**Authors:** Krzysztof Benc, Ewa Tabaka, Wiktoria Pabian, Dominika Pisarek, Karolina Marek-Bukowiec, Andrzej Konieczny, Mirosław Banasik

**Affiliations:** Department of Nephrology, Transplantation Medicine and Internal Diseases, Institute of Internal Diseases, Wroclaw Medical University, 50-556 Wroclaw, Poland

**Keywords:** lupus nephritis, urinary exosomes, biomarkers, microRNA, tsRNA, renal fibrosis, systemic lupus erythematosus

## Abstract

Lupus nephritis is one of the most severe manifestations of systemic lupus erythematosus and remains a major cause of chronic kidney damage and progression to end-stage renal disease. Although kidney biopsy is considered the diagnostic gold standard, its invasive nature, risk of complications, sampling limitations, and limited suitability for repeated monitoring highlight the need for reliable non-invasive biomarkers. Urinary exosomes have emerged as a promising source of renal molecular information because they are easily accessible and contain stable bioactive molecules, including microRNAs, transfer RNA-derived fragments, messenger RNAs, long non-coding RNAs, and proteins. This review summarizes available evidence on urinary exosomal biomarkers in lupus nephritis, with particular emphasis on their potential utility in diagnosis, assessment of disease activity, monitoring of treatment response, and prognosis. The literature search was conducted in PubMed and Embase using the keywords “lupus nephritis” and “urinary exosomes”. Studies were included if they investigated urinary exosomes in lupus nephritis and evaluated their relevance to disease diagnosis, activity, therapeutic response, histopathological findings, or renal outcomes. Among the analyzed biomarkers, urinary exosomal miR-146a appears to be the most consistently reported diagnostic marker, showing increased expression in active lupus nephritis and the ability to distinguish patients with renal involvement from those without nephritis. Other molecules, including selected miRNAs, tsRNAs, long RNAs, and proteins, have also shown potential clinical relevance, although their validation remains limited. Current evidence suggests that urinary exosomes may provide a non-invasive platform for improving the clinical assessment of lupus nephritis. However, most available studies are limited by small sample sizes, single-center designs, heterogeneous methodologies, and insufficient external validation. Further large-scale prospective studies are required to standardize exosome isolation and biomarker quantification and to determine whether urinary exosomal biomarker panels can complement kidney biopsy in clinical practice.

## 1. Introduction

Systemic lupus erythematosus (SLE) is a chronic inflammatory autoimmune disease characterized by a wide range of clinical manifestations. SLE can affect multiple organs simultaneously, including the skin, joints, kidneys, lungs, brain and blood vessels [1]. According to epidemiological data, approximately 400,000 new cases are diagnosed each year, whilst the total number of affected patients globally is estimated at nearly 3.4 million [2,3].

A large systematic review from 2022 revealed that the countries with the highest incidence of SLE include China (8.57, 8.37 to 8.77 per 100,000 person-years), Barbados (10.37, 2.01 to 36.46 per 100,000 person-years), the United States (12.13, 11.94 to 12.35 per 100,000 person-years) and certain European countries, including Poland (81.84, 80.33 to 83.51 per 100,000 person-years) [2].

One of the most severe manifestations of SLE is lupus nephritis (LN), which occurs in approximately 25–60% of patients, depending on the population studied [4,5]. In 10–30% of patients diagnosed with LN, end-stage renal disease (ESRD) develops within 10 years of diagnosis [6]. These data indicate that LN is one of the most significant clinical problems in patients with SLE, which emphasizes the critical importance of its early and effective diagnosis and regular monitoring of the treatment. Patients with SLE require active and regular monitoring, as renal involvement may remain asymptomatic or present with minimal clinical symptoms for a long time [7]. According to the 2024 Kidney Disease Improving Global Outcomes (KDIGO) guidelines, a holistic approach is recommended, involving regular assessment of urine, laboratory markers and clinical data, to identify trends over time that may indicate the development or exacerbation of LN [7]. Given that non-invasive clinical parameters do not always correlate with actual disease progression and demonstrate limited sensitivity and specificity, in assessing the histological activity of LN [8,9,10,11,12,13]. Renal biopsy with histopathological assessment of the tissue is considered a gold standard diagnostic tool [7]. The ISN/RPS histopathological classification divides LN into classes I–VI, and classification into a specific class is of utmost importance for the choice of therapeutic strategy and the assessment of prognosis. However, renal biopsy remains an invasive and time-consuming procedure, carrying a risk of complications, sampling error and subjectivity in histopathological assessment [14,15], which highlights the need to seek less invasive, faster and reliable diagnostic tools for the detection and monitoring of LN.

Among potential, non-invasive sources of biomarkers, exosomes are of particular interest. Thanks to their ability to carry molecular information, reflecting the state of cells and tissues, they represent a promising diagnostic platform for the detection and monitoring of kidney diseases, including LN.

Exosomes are small (30–150 nm), bilayer extracellular vesicles with a phospholipid structure, released by virtually all cell types in the body as functional carriers of molecular information [16]. Initially, they were thought to be structures primarily responsible for the removal of waste products. However, further research has shown that exosomes are important mediators of molecular communication, involved in the transport of proteins, lipids, metabolites and various nucleic acids [17,18]. It has been demonstrated that they are present in numerous body fluids and secretions, such as blood, breast milk, urine, cerebrospinal fluid, saliva and bile [19]. Exosomes are involved in both physiological processes and the pathogenesis of many diseases, playing a significant role in, amongst other things, intercellular communication [20], the immune response [21] and tumor progression [22]. As mentioned above, exosomes contain numerous bioactive substances, including nucleic acids carrying genetic information, encoded on carriers in the form of mRNA and non-coding nucleic acids (RNA), including miRNA and tsRNA [23]. This knowledge has laid the foundations for research into specific molecules, regarding their impact on the signaling pathways involved in the initiation and progression of LN, which may lead to the development of standardized biomarker panels, to aid the evaluation of the disease in patients.

miRNAs are non-coding, small RNAs, approximately 22 nucleotides in length, which mediate post-transcriptional gene regulation [24]. They are the most common type of RNA transported in exosomes [25]. Recent reports suggest a link between the regulation of exosomal miRNA levels and histological changes, suggesting the use of exosomal miRNAs, detected in urine, as biomarkers of renal disease activity and renal fibrosis in LN [26,27].

In turn, tsRNAs are non-coding small RNAs (14–40 nt in length) derived from mature tRNAs or their precursors, which are produced under conditions of enzymatic lysis and cellular stress [28]. tsRNAs can be divided into two categories—tiRNAs (tRNA halves) and tRFs (tRNA-derived fragments) [28]. These molecules have been found to be stable in exosomes circulating in body fluids, including urine [28]. Furthermore, tsRNAs play a significant role in the pathophysiology of biological processes by binding to proteins or mRNA, regulating gene expression and inhibiting translation [29]. Recently, there have been an increasing number of reports on the role of tsRNA in the pathogenesis of SLE [30,31,32,33]. This knowledge enables the use of exosomes as potential diagnostic or therapeutic markers, or as tools for monitoring disease progression. There are numerous scientific studies focusing on the analysis of exosomes extracted from blood [34,35,36,37,38]. However, it appears that in the case of kidney diseases such as LN, exosomes analyzed in patients’ urine would be of greater utility. Unlike testing other tissues, such as blood, urine analysis is a truly non-invasive method. Furthermore, urine is produced near the site of renal disease activity, which offers a promising opportunity for monitoring patients with SLE [39]. This review examines the premise that urinary exosomal biomarkers may represent a valuable non-invasive tool for the diagnosis, assessment of disease activity, treatment monitoring, and prognosis of lupus nephritis.

## 2. Methods

This manuscript was prepared as a narrative literature review focused on urinary exosomes in lupus nephritis. A literature search was performed in PubMed and Embase up to June 2025. The search included combinations of the following keywords: “lupus nephritis”, “systemic lupus erythematosus”, “urinary exosomes”, “urinary extracellular vesicles”, “exosomes”, “extracellular vesicles”, “exosomal miRNA”, “microRNA”, “tsRNA”, “tRNA-derived fragments”, and “long RNA”. Boolean operators (AND/OR) were used as appropriate for each database. Reference lists of eligible articles were also manually screened to identify additional relevant studies. Publications were considered eligible if they investigated urinary exosomes in LN and addressed at least one of the following areas: diagnosis, assessment of disease activity, monitoring of treatment response, histopathological findings, renal fibrosis, or prognosis. Original research articles and review papers written in English were included. Conference abstracts, editorials, letters, and studies outside the scope of the review were excluded.

Following duplicate removal, title and abstract screening, and full-text eligibility assessment, 35 studies met the inclusion criteria. These included 18 original studies forming the basis of the qualitative analysis, while review articles were used to provide background context and support the discussion. The final synthesis included experimental, clinical, observational, and animal model studies.

For each included study, the extracted information included the biomarker type, direction of expression change in urinary exosomes, potential clinical applicability, association with disease activity or histopathological findings, and available diagnostic or prognostic performance parameters, including sensitivity, specificity, and AUC values. Particular attention was paid to the reproducibility of findings across studies, discrepancies between studies, and the degree of evidence supporting the potential clinical utility of each biomarker. Because of methodological heterogeneity and the limited number of studies available for several biomarkers, no meta-analysis was performed.

This review has several limitations. First, the available evidence is derived largely from small, single-center studies, which limits the generalizability of the findings. Second, the included studies used heterogeneous methods for urine collection, exosome isolation, biomarker quantification, and normalization, making direct comparison between studies difficult. Third, several candidate biomarkers have been reported in only one or a small number of cohorts and therefore require external validation. Fourth, the available data do not yet allow a reliable comparison between individual biomarkers and multimarker panels across different clinical settings. Finally, because this article was designed as a narrative review, the findings should be interpreted as a synthesis of current evidence rather than as a quantitative estimate of diagnostic or prognostic performance.

## 3. Results and Discussion

### 3.1. Urinary Exosomal Biomarkers in Lupus Nephritis

To organize the collected data and provide an overview of the reviewed studies, all analyzed biomarkers were summarized in tabular form. Table 1 presents 31 molecules, including miRNAs, mRNAs, lncRNAs, tsRNAs and proteins, together with their potential utility in differentiating clinical conditions and the direction of changes in their concentrations in urinary exosomes in LN. A detailed characterization of each biomarker, along with discussion of the corresponding study findings, from which these data were derived, is provided in the following sections of this review.

### 3.2. Diagnostic Biomarkers

Identifying a biomarker that could support non-invasive diagnosis of LN would be clinically relevant, particularly in view of the limitations of the current gold standard, kidney biopsy. After analyzing studies investigating urinary exosomes, it was observed that the isolated molecules with diagnostic potential belonged to two groups—predominantly miRNAs and, to a lesser extent, tsRNAs [7].

The best documented diagnostic biomarker of LN, among the analyzed studies, is miR-146a, which demonstrated high reproducibility across different research cohorts. One of the earliest studies evaluating the significance of miRNAs in SLE, conducted by Wang et al. (2010) [40], analyzed the levels of miR-146a and miR-155 in serum and urinary supernatants obtained from 40 SLE patients and 30 healthy individuals. This study was particularly important because it demonstrated a distinct expression profile of miR-146a depending on the biological material analyzed. Compared with the control group, serum miR-146a levels were significantly decreased, whereas its urinary levels were elevated in patients with SLE. Perez-Hernandez et al. in 2015 [41] demonstrated that, among the analyzed molecules, miR-146a produced the most promising results. Patients with LN exhibited, on average, a 15-fold higher level of miR-146a than patients with SLE without renal involvement (AUC = 0.960, sensitivity 100%, specificity 90%). Moreover, this marker enabled differentiation between active LN and LN in remission (AUC = 0.867, sensitivity 80%, specificity 89%). In some patients with active LN, miR-146a expression was even 100-fold higher than in the control group. MiR-146a was further evaluated as a marker of LN activity and a predictor of disease flares (Perez-Hernandez et al. 2021) [42]. A cohort of 41 patients with SLE was included, comprising 17 patients with active LN, 10 with LN in remission, and 14 without renal involvement, together with 20 healthy volunteers. It was demonstrated that urinary exosomal miR-146a levels were significantly higher in patients with active LN compared with patients in remission and those with SLE without renal involvement. This marker showed high diagnostic value, with an AUC of 0.82 for LN diagnosis and an AUC of 0.99 for identifying patients with renal involvement.

Increased expression of several additional miRNAs has also been observed, although their diagnostic performance was less promising when assessed individually and they may rather be considered as components of a multi-marker diagnostic panel (Perez-Hernandez et al., 2015) [41]. MiR-335 showed increased expression in urinary exosomes of patients with active LN, reaching approximately a 20-fold increase compared with controls (AUC = 0.822). MiR-200c and miR-302d were elevated in patients with SLE; however, they were unable to differentiate patients with LN from those without renal involvement. These markers are considered nonspecific for kidney injury and their potential utility in LN diagnostics remains limited.

Recently, several studies have investigated the relationship between tsRNAs and SLE [30,31,32,33]. Chen et al. identified two potential biomarkers, tRF3-Ile-AAT-1 and tiRNA5-Lys-CTT, which may serve as markers distinguishing LN-negative SLE patients from LN-positive individuals [39]. The study, involving 173 patients, was divided into three phases: the discovery phase, training phase, and validation phase. Both markers showed higher expression levels in patients with LN compared with SLE patients without renal involvement. Analyzed as independent biomarkers, tRF3-Ile-AAT-1 demonstrated an AUC of 0.777, with a sensitivity of 79.6% and specificity of 66.7%, whereas tiRNA5-Lys-CTT-1 achieved an AUC of 0.715, sensitivity of 67.0%, and specificity of 76.9%. These markers showed substantially greater diagnostic value when combined with other parameters of kidney injury, such as eGFR and albumin levels. A diagnostic panel containing both tsRNAs together with albumin achieved an AUC of 0.881, with a sensitivity of 83.7% and specificity of 94.2%.

Ceruloplasmin (CP) was proposed as a potential non-invasive biomarker of kidney injury Gudehithlu et al. (2019) [43]. Patients with various biopsy-confirmed kidney diseases were included, comprising lupus nephritis (LN)—10 patients, membranous nephropathy (MN)—9 patients, focal segmental glomerulosclerosis (FSGS)—10 patients and IgA nephropathy—7 patients, together with 15 healthy individuals serving as the control group. An animal model was also used in the study, involving rats with Passive Heymann nephritis (PHN), an experimental equivalent of membranous nephropathy. The level of ceruloplasmin in urinary exosomes was found to be 10–20 times higher in patients with CKD than in the control group. However, no significant differences were observed between patients with different underlying causes of kidney disease, indicating that CP is not specific for LN. In the animal model, increased ceruloplasmin levels were detected even before the onset of proteinuria. These findings suggest that ceruloplasmin may be used as an early marker of CKD progression and potentially as a marker of renal involvement risk in patients with systemic diseases such as SLE.

Decreased urinary exosomal levels of miR-195-5p, miR-25-3p, and miR-429 have also been identified as potential biomarkers in a cohort of 47 patients (Cheng et al., 2022) [44]. Among these molecules, miR-195-5p demonstrated the best sensitivity and specificity in differentiating patients with LN from patients with SLE without renal involvement. Although it was considered the most promising miRNA among those analyzed, these findings have not yet been confirmed in further studies.

The potential utility of total urinary extracellular vesicle (uEV) concentration and size distribution as biomarkers of LN has also been evaluated (Navarro-Hernandez et al., 2024) [45]. A cohort of 15 patients with LN and 11 patients with SLE without renal involvement was included. Urine samples were collected within a maximum of 3 weeks after kidney biopsy, allowing direct comparison of the results with current histopathological findings. Total uEV concentration was significantly higher in patients with LN compared with patients with SLE without nephropathy. In particular, the 0.3 μm and 0.5 μm fractions (microvesicles) showed the best ability to differentiate patients with LN from those without renal involvement. For the 0.3 μm fraction, the concentration was 0.80 EV/μL in patients with SLE without renal involvement and 7.41 EV/μL in patients with LN. For the 0.5 μm fraction, the concentration was 0.38 EV/μL in patients with SLE without renal involvement and 1.44 EV/μL in patients with LN. More recently, Alves et al. (2025) [46] further expanded these findings by demonstrating that podocyte-derived uEVs were significantly increased in patients with active LN compared with patients with active SLE without renal involvement. Moreover, podocyte-derived uEVs correlated with proteinuria, albuminuria, and renal disease activity. While uEVs alone showed only modest diagnostic performance, their combination with urinary inflammatory mediators (IL-6, IFN-γ, IL-8, CCL-2, and CCL-3) achieved an AUC of 0.88, suggesting that multimarker panels combining extracellular vesicles and immune mediators may provide greater diagnostic accuracy than individual biomarkers.

### 3.3. Biomarkers of Disease Activity, Treatment Response, and Prognosis

Certain molecules appear to have potential distinguishing whether LN is in the active phase of the disease or in remission.

The study by Heng Zhang et al. focuses on biomarkers obtained from exosomes in urine, which include mRNA molecules: *CCR7*, *LRRN3*, and long non-coding RNAs—lncRNAs: RUNDC3A-AS1 and LINC01127 [47]. Initially, the study included 40 patients, comprising 27 with active LN and 13 with LN in remission, from whom the above-mentioned molecules were isolated. Subsequently, a validation cohort study involved 143 SLE patients, including 89 with active LN, 41 with LN in remission, and 13 with SLE without renal involvement. Expression analysis showed that the concentration of *CCR7* and *LRRN3* biomarkers was significantly lower in active LN compared to the rest of the patients. In contrast, *RUNDC3A-AS1* and *LINC01127* were elevated in active LN; additionally, *LINC01127* levels were lower in patients in remission compared to SLE patients without LN.

Furthermore, the levels of the molecules studied were also compared with a group of healthy individuals. No significant difference was found for *CCR7* and *LRRN3* in healthy individuals compared to patients with LN in remission and non-LN SLE. However, *RUNDC3A-AS1* expression was elevated in patients with active LN and non-LN SLE compared to the control group. Considering the ROC, all four biomarkers were able to distinguish active disease from remission. However, the combination of *LINC01127*, *RUNDC3A-AS1*, and *LRRN3* demonstrated the highest diagnostic performance (AUC = 0.926) with a sensitivity of 89.66% and a specificity of 92.30%. All molecules were correlated with CI, with LRRN3 showing a negative correlation, while *RUNDC3A-AS1* and *LINC01127* showed positive correlations. Furthermore, *RUNDC3A-AS1* was significantly elevated and *LRRN3* significantly decreased in proliferative LN (grade III/IV) compared to isolated membranous LN, suggesting a potential subtyping tool for histological classification of lupus glomerulopathy (AUC = 0.9565, sensitivity 100%, specificity 95.65%).

These data suggest the potential utility of the analyzed markers as indicators of disease status, including differentiation between active and remitted disease, as well as histological classification.

Tangtanatakul et al. investigated let-7a, miR-21, miR-10a, and miR-10b in the context of distinguishing patients with active and inactive lupus nephritis [48]. Initially, urine samples were analyzed from 3 patients during the active phase of LN and again 4 months after therapy, with a positive response. Subsequently, a validation cohort included 13 patients with disease flares and 18 patients in remission. The study demonstrated decreased expression of let-7a and miR-21 in patients with active disease, whereas miR-10a and miR-10b showed inconsistent results among the participants. However, none of the biomarkers showed a significant correlation with renal function parameters such as GFR, serum creatinine, nor proteinuria.

Urinary exosomes are also being studied as a potential tool for monitoring treatment response in patients with LN. Owing to their non-invasive nature and ability to reflect ongoing renal processes, they may provide important support in evaluating therapeutic efficacy and optimizing individualized treatment strategies.

Garcia-Vives et al. conducted a screening cohort study involving 14 patients with proliferative LN (including 7 responders and 7 non-responders), which identified 15 miRNAs with abs(logFC) < 10 and *p* < 0.05 [49]. A validation cohort study was subsequently established, including an additional 22 responders and 21 non-responders. Three molecules were identified: miR-31, miR-107, and miR-135b-5p. A significant increase in the expression of miR-31-5p and miR-107 was observed in the responder group, comparing levels during the active phase of the disease and 12 months after treatment. MiR-135b-5p increased with decreasing proteinuria, in both groups, although a stronger correlation was observed among non-responders. The study found that miR-135b-5p demonstrated the best ROC profile for distinguishing patients responding to treatment from those non-responders, both during disease activity (AUC = 0.783) and after treatment (AUC = 0.855). Additionally, miR-135b-5p differs in that, unlike the other two molecules, it is present not only in tubular cells but also in glomeruli. The common target genes of all three RNA molecules are *HIF1A*, *FOXO1*, and *KLF4*; however, only HIF1A protein levels differed between the responder and non-responder groups. Inhibition of HIF1A expression reduces mesangial cell proliferation and the production of inflammatory cytokines such as IL-6 and IL-8, as well as the chemokines including CXCL1, CCL2, and CCL3. Therefore, reduced HIF1A protein levels may be associated with renal repair processes. Considering the characteristics described by Garcia-Vives et al., miR-135b-5p, miR-31 and miR-107 appear to be the most promising indicators for monitoring therapeutic response in patients with LN.

Appropriate monitoring of LN patients is essential for selecting optimal treatment and timely response to potential disease progression. Renal involvement is often clinically silent and may remain undetected without regular assessment of renal status. Reliable evaluation of disease progression frequently requires repeat biopsy [50]. Identification of a biomarker that reliably reflects LN progression might reduce the need for invasive diagnostic procedures and minimize patient exposure to repeat biopsies. The most promising markers of renal disease progression include miR-29c, miR-21, and miR-150.

MiR-29c has been identified as one of the most promising biomarkers of LN. A 2015 study included 32 patients with biopsy-proven LN, 20 patients with other CKD, and 15 healthy volunteers (Solé et al., 2015) [51]. It demonstrated a clear negative correlation between miR-29c levels and the severity of renal fibrotic changes (Solé et al., 2015) [51]. However, no significant correlation was observed between miR-29c levels and parameters such as eGFR or serum creatinine. Expression of miR-29c reliably predicted the degree of chronicity in patients with LN (AUC = 0.946; *p* < 0.001), with high sensitivity (94%) and specificity (82%). These findings suggest that miR-29c may represent an early, non-invasive biomarker of renal fibrosis, potentially preceding deterioration in renal function. Lower miR-29c levels in patients with more advanced renal lesions, including tubular atrophy, interstitial fibrosis, and glomerulosclerosis, were subsequently confirmed by Solé et al. (2019) [26]. Additionally, miR-21 and miR-150 were identified as markers whose expression increased with the severity of renal damage.

The previously mentioned miR-21 shows seemingly contradictory expression patterns across the analyzed studies. Significantly elevated urinary exosomal miR-21 levels were observed in patients with more advanced renal fibrosis (AUC = 0.742) (Solé et al., 2019) [26]. In contrast, decreased miR-21 levels were reported during renal flares, i.e., in the phase dominated by acute inflammatory activity (Tangtanatakul et al., 2019) [48]. Taken together, these findings suggest that miR-21 may reflect the transition of disease from active inflammation to chronic damage. During the acute phase, when immune activation and inflammatory injury predominate, miR-21 levels appear reduced, whereas with the resolution of inflammation and the emergence of repair processes that may promote pathological fibrosis, miR-21 expression increases. Clinically, this pattern suggests that miR-21 should not be interpreted as a marker of disease activity but rather as an indicator of long-term renal remodeling and the risk of progression toward fibrosis.

miR-150 has also been identified as an important biomarker of fibrosis, showing significant upregulation in patients with severe renal fibrosis (AUC = 0.970) (Solé et al., 2019) [26]. However, no significant correlation was observed with renal function parameters such as serum creatinine and eGFR.

Additionally, a multimarker panel comprising miR-29c, miR-150, and miR-21 demonstrated improved diagnostic performance compared with individual markers, with higher sensitivity and specificity (94.4% and 99.8%, respectively; AUC = 0.996) (Solé et al., 2019) [26]. Its prognostic performance was superior to standard clinical parameters such as serum creatinine or eGFR, suggesting potential utility as a diagnostic tool for renal fibrosis and a prognostic indicator of progression to end-stage renal disease. However, these findings require validation in larger patient cohorts.

Cardenas-Gonzalez et al. (2017) [52] included 89 patients with LN and 119 healthy controls and demonstrated that miR-3201 and miR-1273e are potential biomarkers associated with the severity of histological changes in LN. No association was observed with renal function parameters. However, both markers were significantly associated with the presence of endocapillary inflammation in the glomeruli. Patients with moderate to severe endocapillary inflammation showed lower urinary levels of miR-3201 and miR-1273e compared with those without such changes or with mild inflammation. Furthermore, reduced levels of miR-3201 and miR-1273e were associated with proliferative LN compared to non-proliferative forms.

MiR-30c-5p showed a moderate correlation with the presence of proteinuria. Additionally, its elevated levels correlated with the occurrence of cellular crescents, acute glomerular injury, and arteriosclerosis (Cardenas-Gonzalez et al., 2017) [52].

In 2014, significant changes in miR-26a expression were demonstrated in both renal tissue and urine of patients with LN (Ichii, et al., 2014) [53]. In an animal model, glomerular miR-26a levels were reduced by approximately 50–70% compared with the control group. A similar pattern was observed in patients with LN, in whom the expression of miR-26a in the glomeruli was significantly lower than in healthy individuals. In contrast, urinary exosomal levels of miR-26a were significantly increased in patients with LN compared with controls, suggesting its release from damaged podocytes. Furthermore, a significant correlation was observed between the reduction in miR-26a expression in renal tissue and the severity of proteinuria.

The previously discussed miR-146a marker also showed potential as a prognostic biomarker in LN. Patients were prospectively followed for 36 months, and higher baseline miR-146a levels were associated with an increased risk of disease flares (AUC = 0.89), with patients exhibiting elevated levels showing up to a seven-fold higher risk of flares (Perez-Hernandez et al., 2015) [41].

The diagnostic performance of the most promising biomarkers identified in the reviewed studies is summarized in Table 2, which presents the reported AUC, sensitivity, and specificity values from individual studies.

### 3.4. Discussion

LN is one of the most common severe manifestations of SLE, affecting 25–60% of patients [4,5]. In accordance with both the 2024 ACR and 2024 KDIGO guidelines, the preferred diagnostic approach is to refer patients with proteinuria > 0.5 g/day for percutaneous renal biopsy [7,54].

However, histologically active disease may not manifest clinically significant proteinuria, indicating the presence of a group of patients who will not be included early enough in the diagnostic pathway described above, and as a result, will not receive treatment in time [55,56,57]. A similar approach is recommended in cases of suspected relapse of LN. However, these recommendations are not strong [54]. Renal biopsy is, nevertheless, an invasive procedure carrying a risk of complications, and it is not always possible to perform it in all patients [14,15]. Considering these limitations, there is a need to identify a non-invasive biomarker that meets the criteria for adequate sensitivity and specificity.

We have analyzed all original studies identified through our literature search that investigated urinary exosomal biomarkers in lupus nephritis. The main molecules constituting the content of exosomes of potential clinical significance are small non-coding RNAs, such as the widely described miRNAs. However, the literature also mentions tsRNAs, the acute-phase protein—ceruloplasmin, and, in the most recent reports, long RNAs. The characteristics that make miRNAs useful, as potential non-invasive markers of kidney disease, are their stability within exosomes detected in urine and their proven ability to influence biological processes within cells, following recognition by receptors or via endocytosis [58]. Furthermore, Perez-Hernandez et al. demonstrated that, in active LN, the quantity of intact miRNA is highest in isolated exosomes, compared to supernatant or cell-free urine [41]. Most reports describe the role of miRNAs as factors involved in the pathogenesis of LN, through their influence on fibrogenesis [51], podocyte damage [42,53], mesangial expansion [59] or the formation of immune deposits [41,60]. The miRNA, most frequently cited, is miR-146a [27,40,41,42,61] and, importantly, this marker demonstrates high repeatability and consistency of results across different research groups and over the course of various years of research. The target genes of miR-146a have been identified, namely *IRAK1* and *TRAF6*. By downregulating these genes, miR-146a may exert a protective effect by reducing inflammation and modulating the TLR4/NF-κB signaling pathway. Pan et al. demonstrated that administering VLPs containing miR-146a to lupus-prone mice resulted in reduced autoantibody production and a decrease in total IgG [61]. It may therefore be concluded that miR-146a has potential not only as a non-invasive marker of LN flare-ups, but also as a therapeutic molecule that suppresses inflammation, by reducing levels of pro-inflammatory cytokines such as IFN-α, IL-1β and IL-6. Another extensively studied miRNA is miR-29c, which has been described as a factor playing a significant role in the development of renal fibrosis [26,51].

TGF-β is an important regulator of miRNAs and, importantly, a key mediator of the fibrotic process in the kidneys, negatively affecting the expression of miR-29c via Smad3 signaling mechanisms [62]. Solé et al. confirmed a correlation between Smad3 expression levels and disease chronicity and demonstrated an inverse relationship with miR-29c levels. miR-21 and miR-150, which have also previously been described as associated with the fibrotic process, also exert their effects via the TGF-β signalling pathway [26]. In a study by Solé et al., the authors were the first to propose the use of a multi-marker panel comprising the assessment of three miRNAs—namely miR-29c, miR-21 and miR-150—due to its higher sensitivity and specificity in assessing the chronicity of the disease in patients with LN flares, compared to the assessment of these markers individually [26]. Its prognostic value was higher than standard markers such as creatinine or eGFR, suggesting that this panel may be used both as a diagnostic marker for renal fibrosis and as a prognostic indicator of progression to end-stage renal failure.

In addition to its influence on the fibrosis process, miR-21 also functions as a mediator of the inflammatory response in macrophages, by inhibiting IL-12 and *PDCD4* [63,64]. There are also reports of its influence on certain genes in the JAK-STAT signaling pathway, which is also associated with the development of inflammation [65]. However, reports concerning miR-21 are inconsistent, with both upregulation [26] and downregulation [48] of this marker being described. An increase in urinary miR-21 levels may result from the accumulation of leukocytes in the urine, by which it is produced [46].

An analysis of the literature has revealed significant discrepancies and variability, as well as a lack of consistency regarding the miRNAs studied, resulting in a multitude of reports on various miRNAs. In most cases, except for those described above, there is a lack of further research that would enable the continued evaluation of specific molecules in clinical practice. Most of the studies conducted are also based on a small study group. It complicates the validation and identification of the best marker or multi-marker panel. A comprehensive discovery study identified several novel urinary miRNAs, including miR-3201, miR-1273e, miR-204-5p, and miR-30c-5p, considerably expanding the spectrum of potential biomarkers for lupus nephritis [52]. However, despite their promising diagnostic performance, these molecules have not yet undergone independent validation, limiting their current clinical applicability. Nevertheless, their reported associations with glomerular inflammation, proteinuria, podocyte homeostasis, and chronic kidney injury suggest that they may provide complementary information on distinct pathological processes involved in LN [52,66]. Similarly, Li et al. identified additional candidate miRNAs associated with crescent formation, supporting the concept that urinary exosomal biomarkers may also reflect histopathological severity rather than disease activity alone [27]. Since cellular crescents are associated with poor renal prognosis and rapid progression to kidney failure [67,68], biomarkers reflecting these lesions may be particularly valuable for risk stratification and treatment monitoring. Recent studies focused on the isolation of molecules other than miRNAs from exosomes as potential markers for LN. Reports on tsRNAs suggest their potential as biomarkers with even greater clinical significance than miRNAs, due to their higher concentration in exosomes [69,70,71], as well as their regulatory role in chromatin status within immune cells, which has a critical impact on the onset and progression of autoimmune diseases [28].

Their reported diagnostic performance and biologically plausible involvement in IGF- and PDGF-related pathways suggest potential utility in both disease assessment and fibrosis progression [71,72,73]. However, these findings originate from a single research group and therefore require independent validation before clinical translation.

Another clinical trial, published in 2025, explored long RNAs [47]. Zhang et al. highlight the advantages of long RNAs over small RNAs, such as greater tissue specificity and ease of detection using standard RT-qPCR, unlike small RNAs, which require complex and costly amplification protocols [47]. The authors identified three candidates, LINC01127, RUNDC3A-AS1 and LRRN3-as, as diagnostic and prognostic markers for LN. Furthermore, RUNDC3A-AS1 and LRRN3-as demonstrated a very strong ability to distinguish between the proliferative (class III/IV) and membranous (class V) types of LN. These markers have been linked to processes of tissue fibrosis [74] and the promotion of pro-inflammatory cytokine expression [75,76]. Interestingly, RUNDC3A-AS1 has been linked to oncogenic pathways, including the miR-182-5p/*ADAM9* axis [77]. *ADAM9* plays a role in the TGF-β signaling pathway and in Th17 cell differentiation, both of which have been linked to the pathophysiology of LN [74,78]. The markers identified as having the greatest potential utility are miR-146a, miR-29c, miR-21, miR-3201, miR-1273e, miR-204-5p and miR-30c-5p. Another interesting clinical direction, though one requiring in-depth analysis by future research groups is tsRNA, and in particular, tiRNA-Lys-CTT-1, and tRF3-Ile AAT-1, as described by Chen et al. [44], as well as long RNAs, including LINC01127, RUNDC3A-AS1 and LRRN3-as.

Beyond individual biomarkers, one of the major limitations of the current evidence is the considerable methodological heterogeneity across studies. As summarized in Table 3, most investigations were conducted in relatively small single-center cohorts and differed substantially with respect to extracellular vesicle isolation methods, biomarker quantification, normalization strategies, and study design. Consequently, direct comparison of biomarker performance across studies remains challenging, and the reproducibility of many promising findings has yet to be established. These methodological differences may contribute as much to the observed variability in biomarker performance as biological differences between patient populations. Therefore, future multicenter studies using standardized pre-analytical protocols, harmonized analytical methods, and consistent normalization strategies will be essential for reliable validation and successful clinical implementation of urinary extracellular vesicle biomarkers. Another valuable line of inquiry appears to be the identification of an appropriate multi-marker panel, which would demonstrate significantly greater sensitivity and specificity than a single marker.

## 4. Conclusions

Urinary exosomes represent a promising non-invasive source of candidate biomarkers for lupus nephritis. Among the molecules reviewed, miR-146a currently appears to have the strongest diagnostic support, whereas miR-29c, miR-21, and miR-150 are particularly relevant to renal fibrosis and chronic histological damage. Emerging biomarker classes, including tsRNAs and long RNAs, may expand the diagnostic and prognostic potential of urinary exosomal analysis. Despite these promising findings, several barriers still limit the clinical translation of urinary exosomal biomarkers. The lack of standardized protocols for urine processing, exosome isolation, RNA extraction, and data normalization hampers the reproducibility and comparability of results across studies. Furthermore, current analytical workflows remain relatively costly, technically demanding, and are not yet routinely available in clinical laboratories. Consequently, although urinary exosomal biomarkers show considerable potential as non-invasive tools, they should currently be regarded as complementary rather than alternative to kidney biopsy until larger validation studies and standardized diagnostic assays become available.

## Figures and Tables

**Table 1:** Summary of biomarkers analyzed in lupus nephritis, including their type and potential clinical significance.

Biomarker	Type	Direction of Change in Urinary Exosomes	Potential Clinical Significance
miR-146a	miRNA	Increased in active LN	Diagnosis of LN, differentiation of active LN from remission, flare risk, association with proteinuria and histological activity
miR-155	miRNA	Reported in SLE urine/serum studies	Exploratory marker evaluated together with miR-146a; clinical specificity for LN remains limited
miR-335	miRNA	Increased in active LN	Potential diagnostic marker; less robust than miR-146a when used alone
miR-200c	miRNA	Increased in SLE	Not specific for renal involvement; limited diagnostic utility for LN
miR-302d	miRNA	Increased in SLE	Not specific for renal involvement; limited diagnostic utility for LN
tRF3-Ile-AAT-1	tsRNA	Increased in LN	Differentiation of SLE patients with LN from those without renal involvement; stronger value in combined panels
tiRNA5-Lys-CTT-1	tsRNA	Increased in LN	Differentiation of SLE patients with LN from those without renal involvement; stronger value in combined panels
Ceruloplasmin	Protein	Increased in urinary exosomes in CKD	Early marker of kidney injury; not specific for LN
miR-195-5p	miRNA	Decreased in LN	Potential diagnostic marker differentiating LN from SLE without renal involvement; requires validation
miR-25-3p	miRNA	Decreased in LN	Potential diagnostic marker; evidence remains preliminary
miR-429	miRNA	Decreased in LN	Potential diagnostic marker; evidence remains preliminary
*CCR7*	mRNA	Decreased in active LN	Differentiation of active LN from remission; associated with chronicity index
*LRRN3*	mRNA	Decreased in active LN	Differentiation of active LN from remission; possible contribution to histological classification
*RUNDC3A-AS1*	lncRNA	Increased in active LN	Assessment of disease activity; association with chronicity index and proliferative LN
*LINC01127*	lncRNA	Increased in active LN	Assessment of disease activity; part of a high-performing multi-marker model
let-7a	miRNA	Decreased in active disease	Potential marker distinguishing active from inactive LN; no clear correlation with renal function parameters
miR-21	miRNA	Decreased in renal flares; increased in advanced fibrosis	Marker potentially reflecting transition from acute inflammation to chronic renal remodeling/fibrosis
miR-10a	miRNA	Inconsistent	Exploratory marker of disease activity; limited standalone utility
miR-10b	miRNA	Inconsistent	Exploratory marker of disease activity; limited standalone utility
miR-31/miR-31-5p	miRNA	Increased in responders after treatment	Potential marker for monitoring therapeutic response
miR-107	miRNA	Increased in responders after treatment	Potential marker for monitoring therapeutic response
miR-135b-5p	miRNA	Changes with proteinuria reduction	Promising marker for distinguishing responders from non-responders
miR-29c	miRNA	Decreased with renal fibrosis/chronicity	Marker of renal fibrosis and chronic histological damage; prognostic relevance
miR-150	miRNA	Increased with severe renal fibrosis	Marker of renal fibrosis and chronic damage; useful in multi-marker fibrosis panels
miR-3201	miRNA	Decreased with moderate/severe endocapillary inflammation	Associated with histological severity and proliferative LN
miR-1273e	miRNA	Decreased with moderate/severe endocapillary inflammation	Associated with histological severity and proliferative LN
miR-30c-5p	miRNA	Increased with proteinuria and acute glomerular lesions	Associated with proteinuria, cellular crescents, acute glomerular injury and arteriosclerosis
miR-26a	miRNA	Increased in urinary exosomes; decreased in renal tissue	Potential marker of podocyte injury and proteinuria-related renal damage
miR-3135b	miRNA	Differentially expressed in LN with crescent formation	Potential marker related to cellular crescent formation; preliminary evidence
miR-654-5p	miRNA	Differentially expressed in LN with crescent formation	Potential marker related to cellular crescent formation; preliminary evidence

**Table 2:** Diagnostic performance of the most promising urinary exosomal biomarkers for lupus nephritis reported across studies.

Biomarker	AUC	Sensitivity	Specificity	Study
miR-146a	0.82–0.99	80–100%	83–90%	Perez-Hernandez et al., 2015 [41] Perez-Hernandez et al., 2021 [42]
miR-29c	0.946	94%	82%	Solé et al., 2015 [51]
miR-21	0.742	81%	72%	Solé et al., 2019 [26]
miR-150	0.970	96%	83%	Solé et al., 2019 [26]
multimarker panel (miR-29c, miR-150, and miR-21)	0.996	94.4%	99.8%	Solé et al., 2019 [26]
tRF3-Ile-AAT-1	0.777	79.6%	66.7%	S. Chen et al. [39]
tiRNA5-Lys-CTT-1	0.715	67.0%	76.9%	S. Chen et al. [39]
multimarker panel (tRF3-Ile-AAT-1, tiRNA5-Lys-CTT-1)	0.881	83.7%	94.2%	S. Chen et al. [39]

**Table 3:** Methodological characteristics of the original studies investigating urinary extracellular vesicle biomarkers in lupus nephritis.

Study	Study Design	Setting	Study Population	Isolation Method	Biomarker Assessment
Ichii et al., 2014 [53]	Translational study including human samples, lupus-prone mice and in vitro experiments	Single-center	*n* = 15	Differential centrifugation and ultracentrifugation	RT-qPCR
Solé et al., 2015 [51]	Cross-sectional study with biopsy correlation	Single-center	*n* = 67	Ultracentrifugation	RT-qPCR
Perez-Hernandez et al., 2015 [41]	Cross-sectional study	Single-center	*n* = 50	Ultracentrifugation	RT-qPCR
Cardenas-Gonzalez et al., 2017 [52]	Discovery and validation study	Single-center	*n* = 152	Total urinary cellular pellet; no isolated EV fraction	Global miRNA profiling followed by RT-qPCR
Li et al., 2018 [27]	Discovery and validation study	Single-center	*n* = 57	Differential ultracentrifugation	Small RNA sequencing followed by RT-qPCR
Tangtanatakul et al., 2019 [48]	Prospective longitudinal study	Single-center	*n* = 31	Ultracentrifugation	RT-qPCR
Solé et al., 2019 [26]	Cross-sectional study with biopsy correlation	Single-center	*n* = 65	Ultracentrifugation	RT-qPCR
Gudehithlu et al., 2019 [43]	Cross-sectional study with complementary longitudinal experiments in an animal model	Single-center	*n* = 51	Differential centrifugation and ultracentrifugation	Immunoblotting and ELISA
Garcia-Vives et al., 2020 [49]	Discovery and validation study	Single-center	*n* = 14	Precipitation-based commercial method	qPCR array followed by RT-qPCR
Perez-Hernandez et al., 2021 [42]	Prospective longitudinal study	Single-center	*n* = 61	Ultracentrifugation	RT-qPCR
Cheng et al., 2022 [44]	Discovery and validation study	Multicenter	*n* = 47	Ultracentrifugation	Bioinformatic miRNA–mRNA network analysis and RT-qPCR
Chen et al., 2023 [36]	Discovery and validation study	Single-center	*n* = 40	Polymer-precipitation method	tsRNA sequencing followed by RT-qPCR
Navarro-Hernandez et al., 2024 [45]	Cross-sectional study	Single-center	*n* = 26	Differential centrifugation	Flow cytometry
Alves et al., 2025 [46]	Cross-sectional study	Single-center	*n* = 100	Differential centrifugation	Flow cytometry and multiplex immunoassay
Zhang et al., 2025 [47]	Discovery and validation study	Multicenter	*n* = 40	Affinity-based magnetic-bead capture	RNA sequencing and RT-qPCR

## Data Availability

No new data were created or analyzed in this study. Data sharing is not applicable to this article.

## Abbreviations

The following abbreviations are used in this manuscript:

RNA	Ribonucleic acid
miRNA	Micro ribonucleic acid
tsRNAs	Transfer RNAs
SLE	Systematic lupus erythematosus
ESRD	End-stage renal disease
LN	Lupus nephritis
KDIGO	Kidney Disease Improving Global Outcomes
CP	Ceruloplasmin
CKD	Chronic renal disease
